# WEIGHT-BEARING COMPUTED TOMOGRAPHY OF THE FOOT AND ANKLE: AN UPDATE AND FUTURE DIRECTIONS

**DOI:** 10.1590/1413-785220182602188482

**Published:** 2018

**Authors:** ALEXANDRE LEME GODOY-SANTOS, CESAR DE CESAR, WEIGHT-BEARING CT INTERNATIONAL STUDY GROUP

**Affiliations:** 1. Instituto de Ortopedia e Traumatologia, Hospital das Clínicas HCFMUSP, Faculdade de Medicina, Universidade de São Paulo, SP, Brazil.; 2. Department of Orthopedics, Hospital for Special Surgery, New York, NY, USA.

**Keywords:** Ankle. Foot. Weight-bearing. Tomography, x-ray computed/methods., Tornozelo, Pé, Suporte de carga, Tomografia computadorizada, imagem/métodos.

## Abstract

Spatial understanding of osteoarticular deformities of the foot and ankle is vital to correct diagnosis and therapeutic decision making. Poor reproducibility in conventional standing radiography in three orthogonal views has driven the development of weight-bearing computed tomography (WBCT) technology over the last decade. We analyzed the available literature on WBCT imaging in patients with foot and ankle disorders by performing a literature review of relevant clinical studies in multiple databases including PubMed, MedLine, and Scopus from January 1999 to October 2017. WBCT imaging allows correct evaluation of foot and ankle anatomy with the patient in a standing position, providing images with high spatial resolution, short image acquisition time, low dose of radiation, and costs which are similar to other available imaging technologies. This diagnostic tool can be used for decision making in the treatment of deformities of the ankle, hindfoot, midfoot, and forefoot. Level of Evidence III; Systematic review of level III studies.

## RESUMO

Na topografia do tornozelo e do pé é fundamental o entendimento espacial das deformidades osteoarticulares para correto diagnóstico e decisão terapêutica. A dificuldade de reprodução da avaliação com radiografias convencionais em posição ortostática em três dimensões impulsionou, na última década, o desenvolvimento da tecnologia de tomografia computadoriza com carga. Analisamos a literatura relacionada com o tema tomografia computadorizada com carga em pacientes com distúrbios do pé e do tornozelo. Para fazer isso, realizamos uma revisão da literatura de estudos clínicos relevantes nas bases de bancos eletrônicos, incluindo PubMed, MedLine e Scopus, de janeiro de 1999 a outubro de 2017. A tomografia computadorizada com carga permite a avaliação da anatomia na posição ortostática fisiológica, com imagens de alta resolução espacial, pequeno tempo de aquisição de imagens, baixa dose de radiação e custos similares a outras tecnologias atualmente disponíveis. Ela pode ser usada para tomada de decisão terapêutica em deformidades do tornozelo, retropé, mesopé e antepé. Nível de Evidência III; Revisão sistemática de estudos de nível III.

## INTRODUCTION

In the area of ankle and foot performance, imaging studies are fundamentally important aids in diagnosis, therapeutic decision-making, and evaluation of functional results. The most commonly used resources are conventional X-rays with load, ultrasound, computed tomography (CT), and magnetic resonance imaging (MRI).^1^

The initial diagnostic investigation often uses conventional X-rays with load to more accurately reproduce the three-dimensional bone relationships in the ankle and foot. However, in many situations the information acquired from this method is limited (especially in relation to the different planes of the ankle and foot) and usually needs to be complemented for correct therapeutic decision making.^1^

The choice of complementary image study is based on certain criteria such as availability, sensitivity, and specificity of the method, cost, and adverse effects/safety, including exposure to radiation.^1^

In this scenario, computed tomography (CT) allows acquisition of high-resolution images in different axes of the ankle and foot, and is usually used to evaluate fractures, degenerative changes, bone healing, and surgical planning for osteotomies, arthrodeses and arthroplasties.^2^

However, a major limit of conventional CT is its inability to reproduce images of feet and ankles subjected to body weight load. In the absence of support for the patient’s body weight, true alignment is not measured correctly. Therefore, this imaging resource is limited, particularly in scenarios related to axial deviations and osteoarticular degeneration such as acquired flatfooted valgus, pes cavus, Charcot’s neuroarthropathy, osteoarticular deformities, diabetic foot, and dynamic ligament instability.^2^

Many researchers have made efforts to develop auxiliary methods to simulate body weight support by the ankle and foot, using different strategies. These authors recognized that the deficiencies in simulated weight support conditions unfortunately did not resolve the limitation of conventional CT.^3^ ^-^ ^10^ Additionally, the devices that simulate body weight load generally utilize passive application of force, have a low standard of reproducibility, and do not permit the active muscle forces that act during orthostatic physiological positioning.^11^ ^-^ ^13^

In this sense, the concept of visualizing the relative alignment of the bones in the ankle and foot using weight-bearing computed tomography (WBCT) is not new. Over the last decade, the cone beam computed tomography with load technique (WBCT) proved feasible and to have high reproducibility of the real situation of the ankle and foot with regard to body weight.^2^ ^,^ ^14^

The advantages of this new technology include: the ability to obtain images with the patient in an orthostatic position, high resolution, possibility of reconstruction in three dimensions, rapid image acquisition, low rate of radiation exposure, small device size, and low cost in relation to conventional CT.^15^

This article presents a review of this important technological innovation in patients with foot and ankle disorders.

### Exposure to radiation and its effects on humans

**Figure 1:**
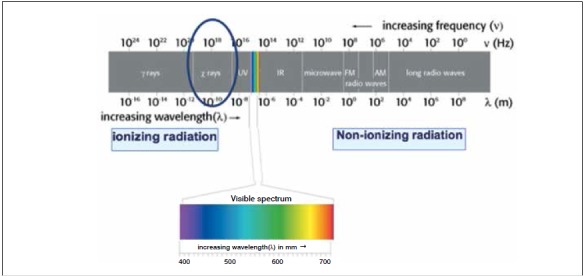
Graph representing the spectra of potential radiation according to frequency in Hertz (Hz) and wave size in Lambda (λ).

Radiation is energy in the form of electromagnetic waves, which can be ionizing or non-ionizing. X-rays are located on the spectrum of ionizing radiation.^16^ ^,^ ^17^ (Figure 1)

The energy produced by X-rays is measured in rems, and the energy deposited in inert materials is measured in grays (Gy), with 1 Gy equivalent to 1 Joule/kg. The energy deposited in living tissue (equivalent dose) is measured in Sieverts (Sv), and 1 Sv is the equivalent of 1 Joule/kg, which reflects the biological effects of ionizing energy.^16^

The somatic and cumulative effects (not determined by dose) of ionizing radiation can result in cancer, genetic mutations, and teratological malformations (at the beginning of pregnancy).^17^

Table 1 shows different sources of ionizing radiation and their respective doses deposited in human tissue in Sieverts.

**Table 1:** Dose of radiation in living tissue by type of human exposure.

Radiation from high-altitude flights	0.001-0.01 mSv/hour
Radiation from natural lighting	0.01 mSv/day
Radiation from simple X-ray of the thorax (anteroposterior)	0.02 mSv
Radiation from simple X-ray of the foot (single exposure)	0.001 mSv
Radiation from surgical radioscopy	0.0375 mSv/3 months
Radiation from surgical radioscopy	0.21 mSv/3 months
Radiation from conventional CT, cranium	1.5 mSv
Radiation from conventional CT, ankle	0.07 mSv
Radiation from conventional CT, full body	9.9 mSv
Weight-bearing CT (WBCT) of the foot/ankle	

Abbreviations: CT, computed tomography; mSV = millisievert.

## STUDIES CONDUCTED PRIOR TO WBCT, USING CT WITH SIMULATED LOAD

### Method using 75 Newton (N) axial force plate, in supine position

In a case-control study with 12 patients (8 with flatfoot valgus and four asymptomatic), Ananthakrisnan et al.^3^ demonstrated less subtalar joint contact in patients with posterior tibial tendon dysfunction (PTTD).

In a case-control study with 24 patients (19 with flatfoot valgus and 5 asymptomatic), Malicky et al.^4^ observed a higher prevalence of lateral impact in the subtalar joint within the tarsal sinus (92% vs. 0%) and calcaneal-fibular joint (66 vs. 5%) in comparison with controls.

Greisberg et al.,^5^ in a case series with 37 patients with PTTD, demonstrated increased deformities in these patients when evaluating the talo-navicular and navicular cuneiform joints, and subluxation of the first tarsal-metatarsal joint.

Apostle et al.,^6^ in a case-control study with 40 patients (20 with peritalar subluxation and 20 healthy volunteers), demonstrated that the subtalar joint axis presents greater valgus in patients with peritalar subluxation.

### Computed tomography using total body weight support platform, in supine position

Geng et al.,^7^ in a case-control study with 20 patients (10 with hallux valgus and 10 healthy volunteers) showed greater dorsiflexion and supination of the first tarsal-metatarsal joint in patients with hallux valgus.

Kido et al.,^8^ in another case-control study with 42 patients (21 with flatfoot valgus and 21 healthy volunteers), observed that patients in the case group had greater plantar talus flexion, navicular abduction in the talo-navicular joint, and calcaneal dorsiflexion and eversion in the subtalar joint when compared to the controls.

Kimura et al.,^9^ in a case-control study with 20 patients (10 with hallux valgus and 10 healthy volunteers) showed greater dorsiflexion in the talo-navicular and first tarsal-metatarsal joints in patients with hallux valgus.

Kido et al.,^10^ in a case-control study with 44 patients (20 with valgus flat foot and 24 healthy volunteers) demonstrated greater dorsiflexion of the first metatarsal, greater eversion of the navicular and the calcaneus, and greater rotation in the talo-navicular joint.

Van Bergeyk et al.,^11^ in a case-control study with 23 patients (11 with chronic lateral instability of the ankle and 12 healthy controls), observed a significantly different hindfoot alignment angle between the groups: 6.4° ± 4º varus in the instability group and 2.7° ± 5° varus in the controls.

Yoshioka et al.,^12^ in a case-control study with 20 patients (10 with flatfoot valgus and 10 healthy volunteers) identified greater forefoot supination in patients with flatfoot valgus.

Zhang et al.,^13^ in a case-control study with 30 patients (15 with flatfoot valgus and 15 healthy volunteers) identified significant differences with regard to the position of the talus, navicular, and calcaneal joint between the groups.

### Studies with WBCT in normal asymptomatic volunteers

Lepojärvi et al.^18^ evaluated the normal anatomy and rotational dynamics of the distal tibiofibular joint in 32 asymptomatic individuals under physiological conditions. Images were acquired at for three different ankle rotations: neutral, internal and external. Four parameters were measured: 1) sagittal translation of the fibula, 2) anterior and posterior width of the syndesmosis, 3) tibiofibular free space, and 4) rotation of the fibula. With the ankle in neutral position, the fibula was seen to be located anterior to the tibial notch in 88% of the volunteers during all the measurements. During rotational movement of the ankle, the mean anteroposterior movement was 1.5 mm and the average rotation of the fibula was 3 degrees.^18^ In the same population, these authors also assessed the rotational dynamics of the talus within the upper section of the ankle joint between the lateral and medial malleoli. When the ankle was turned with strength equivalent to 30 Nm, a 10° rotation was observed without a substantial increase in free medial space.^19^

Cody et al.^20^ performed WBCT in 59 volunteers without a history of previous disease or foot/ankle injury to describe their findings in the subtalar joint. The orientation of the posterior facet of the subtalar joint was measured in three different coronal planes (at the center of the subtalar joint, and 5 mm anterior and posterior to the center). These authors observed a concave posterior facet in 88% of the volunteers and flat facet in the other 12%. In the coronal plane, the posterior facet was in valgus in 90% of the images and in varus in the other 10%. They also found greater valgus angulation in positions more posterior to the subtalar joint.

### WBCT studies in patients with deformities

A total of 12 studies published between 2001 and 2017 were selected: two case reports, five prospective studies, and five retrospective studies. The levels of evidence ranged from II to IV, with two level II studies, six level III studies, and four level IV studies.

## CASE REPORTS

Welck and Meyerson^21^ described an unusual case of bilateral atraumatic erosive subtalar osteoarthritis with unilateral subtalar collapse, and used WBCT for surgical planning and postoperative evaluation. These authors emphasized the value of this method in pre-surgical planning, since it allowed the relevant angles to be measured precisely in three dimensions, exactly determining the presence of posterior osteophytes and anterior and lateral impact in the ankle. They also emphasized its use in postoperative follow-up, permitting a functional and anatomically correct assessment of the correction performed.

Using Kaplan’s analysis, these same authors described their findings from a study using WBCT in three cases of Muller-Weiss disease.^22^

## CASE REPORTS

Burssens et al.^15^ described a clinically reproducible method for measuring hindfoot alignment using WBCT. In a prospective case series with 60 patients divided into two groups (30 patients with varus alignment and 30 patients with valgus hindfoot alignment), these authors observed a positive correlation between the hindfoot alignment angles measured and concluded that WBCT can be used objectively for this measurement.

### Tomography studies with partial load

Kim et al.^23^ used CT with partial load to evaluate preoperative alignment of the forefoot in 138 patients (166 feet) with hallux valgus deformities and compared their results with a control group of 19 patients (19 feet). These authors evaluated the angle α (pronation angle of the first metatarsal) and the relative position of the sesamoids. Angle α and subluxation of the sesamoids differed significantly between the study group and the control group. The authors suggested that the use of CT with partial load might be useful in assessing the deformity of the forefoot in the coronal plane and guiding the choice of treatment of patients with hallux valgus.

### Case-control studies using CT with load

Cody et al.^20^ used WBCT to analyze the anatomy of the talus and the alignment of the subtalar joint in 45 patients with adult type II acquired flatfoot and 17 volunteer controls. The subtalar alignment was assessed using the angles between the bottom facet of the talus and the ground and the angle between the upper and lower facets of the talus. Both of these angles were seen to differ significantly between the study groups. The researchers concluded that patients with flatfoot valgus deformity presented greater innate valgus in their talar anatomy and greater alignment of the subtalar joint in valgus. They emphasized that these measures can be used to identify patients with higher risk of progressive deformity and subtalar joint degeneration.

Krähenbühl et al.^24^ analyzed subtalar orientation using WBCT in 40 patients with tibiotalar osteoarthritis and 20 healthy controls. Subtalar alignment was assessed through the angle between a line perpendicular to the ground and the posterior facet of the subtalar joint. When they compared the joints in varus and valgus, the authors observed significant differences when compared to healthy controls. They concluded that the orientation of the subtalar joint could be a determinant factor in the development of ankle osteoarthritis.

Lintz et al.^25^ described a new three-dimensional biometric tool for WBCT to evaluate hindfoot alignment, using the concept of the biomechanical tripod formed by the head of the first and fifth metatarsals and the farthest point of the calcaneal tuberosity in relation to the positioning of the center of the ankle joint, represented by the point closest to the domus talar. This relationship is represented by the foot-angle offset (FAO). The data set from the population studied was analyzed (57 volunteers with normal hindfoot alignment, 38 volunteers with varus alignment, and 40 volunteers with valgus alignment), and the authors observed FAO of 2.3% ± 2.9% in the controls, -11.6% ± 6.9% in the patients with varus of the hindfoot, and 11.4% ± 5.7% in patients with hindfoot valgus. They concluded that the method described was feasible and reproducible for measuring foot-ankle offset and hindfoot alignment.^25^

In a prospective study of 50 patients with symptomatic hallux rigidus and 50 controls who underwent CT with load assessed by two examiners in relation to the difference in length between the first and second metatarsals, intermetatarsal angle between the first and second metatarsals (IMA), and hallux valgus angle (HVA), Cheung et al. observed a smaller difference in length, smaller IMA, and smaller HVA in the patients with hallux rigidus than in the controls.^26^

### Studies comparing CT with load and pedobarography

In a prospective study, Richter et al.^27^ evaluated 50 patients who simultaneously underwent WBCT and pedobarography. The authors mapped the alignment of the hindfoot and midfoot, and the relationship between the head of the first metatarsal/sesamoids and the heads of the lateral metatarsals (2nd, 3rd, 4th, and 5th) and with all the toes (1st-5th). These authors found no significant correlation between bone alignment measurements in WBCT and the distribution values for plantar pressure in pedobarography.

### Studies comparing CT with load and simple X-ray with load

Kim et al.^14^ evaluated conventional X-rays with load and WBCT for 96 patients with osteoarthritis (OA) of the ankle, divided into groups with moderate OA (50 patients) and severe OA (46 patients). These authors documented the presence of abnormal internal rotation of the talus in patients with osteoarthritis in varus, which was more frequently observed in the group with severe OA than those with moderate OA. They emphasized that this rotation could not be noted in conventional X-rays since axial images cannot be acquired.

### Studies comparing CT with and without load

Collan et al.^28^ compared the alignment of the first metatarsal in 10 patients with hallux valgus with five asymptomatic controls using CT with and without load; these authors observed an increase in medial deviation of the first metatarsal and pronation of the first toe on images with load in patients with hallux valgus.

Hirschmann et al.^29^ performed a prospective evaluation of multiple alignment measurements in 22 volunteers using CT with and without load. These authors found significant differences for most measurements: distance between fibula and calcaneum, lateral subtalar joint space, talus-calcaneus overlap, and calcaneus-navicular distance. They found no difference between the hindfoot alignment angle and distance between the tibia and calcaneus when comparing images from the group with load and without load. The hindfoot alignment angle was comparable when measured with and without load (21.0° ± 7.9° vs. 19.0° ± 9.0°). The authors suggested using WBCT in assessing fibular impact and talus-calcaneus overlap.

Richter et al.^30^ prospectively evaluated foot and hindfoot alignment in 30 patients using WBCT, CT without load, and conventional X-rays with load. They found significant differences in angles measured using different imaging methods. The hindfoot alignment angle in the WBCT was 10.1º ± 7.16, 5.4º±5.6º in CT without load, and 2.4º±6.9º in conventional X-ray with load.

de Cesar Netto et al.^2^ prospectively evaluated multiple parameters used in measuring adult valgus flatfoot deformity, comparing CT images with and without load from 20 patients diagnosed with flexible deformity. These authors demonstrated that WBTC produced similar measurements to those traditionally obtained from conventional X-ray imaging to stage adult valgus flatfoot deformity. They also noted that the measures, which indicate the severity of the deformity, are more pronounced in images obtained with load than those obtained without load.

## FINAL CONSIDERATIONS

Computed tomography with load is available to investigate osteoarticular deformities of the ankle and foot. This method allows more suitable and reliable assessment of the anatomy in a physiological position with load, closer to the mechanical demands of normal gait. This technique provides images with high spatial resolution, with rapid image acquisition, low radiation dose, and costs similar to other technologies available. WBCT may be used for therapeutic decision making in deformities of the ankle, hindfoot, forefoot, and midfoot, to help determine more accurate surgical planning. 
